# Supplementary material for: Comparison of Two Field Deployable PCR Platforms for SARS-CoV-2 and Influenza A and B Viruses’ Detection
Source: Pathogens. 2025 Jan 3;14(1):27. doi: 10.3390/pathogens14010027 (PMC11768418; doi:10.3390/pathogens14010027)
Supplement: Supplementary file 1 [file pathogens-14-00027-s001.zip › pathogens-3359343-supplementary.pdf]

Diagnostic platform	**Biomeme Franklin™** 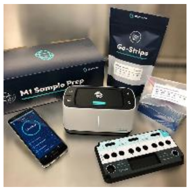 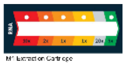	**Truelab®** 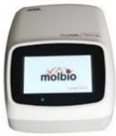 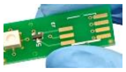
Website links for updated information	https://biomeme.com/platforms/franklin	https://www.molbiodiagnostics.com/product_details.php?id=24<br>https://www.molbiodiagnostics.com/product_details.php?id=57
Initial Cost of equipment	~$16,000 USD per system	~$18,000 USD per system
Test Kit Cost (per test)	~$12 USD per test	~$13 USD per test for FluA/B<br>~$15 USD per test for SARS-CoV-2
Reagents	Biomeme integrated M1 RNA extraction cartridge	Molbio Integrated RNA extraction module
RNA Extraction Cost	~$30-50 USD per extraction (depending on scale)	Integrated with machine, no additional cost
Consumables per Run	Low cost per run (~$1-3 for consumables)	Includes consumables with test kit (~$3-5)
Maintenance Cost	Low (few moving parts, minimal maintenance)	Moderate (requires regular servicing)
Operational Time	~45 minutes per run	~60 minutes per run
Sample Capacity	9 targets in triplicate or 27 individual targets per run	1-4 samples per run (depending on setup)
Failure Rate	Lot # number dependent	~0.5%
Platform Usability	Point-of-care, compact, portable	Point-of-care, compact, portable
Flexibility	No multiplexing capability	No multiplexing capability
Deployment Feasibility	Easy to deploy; suitable for small teams or units	Slightly less portable, but can handle more complex scenarios
Battery Life	6 hours per full charge	5-6 hours per full charge
Data Handling	Cloud-based data management	Integrated with device for real-time results

Supplementary Table S1: Usability and cost effectiveness comparative analysis between Biomeme Franklin^{TM} and Truelab® .
